# Case series of the first three severe COVID-19 patients treated with the secretome of hypoxia-mesenchymal stem cells in Indonesia

**DOI:** 10.12688/f1000research.51191.2

**Published:** 2021-07-22

**Authors:** Agung Putra, Agus Widyatmoko, Sugeng Ibrahim, Fajar Amansyah, Farid Amansyah, Mukti Arja Berlian, Retnaningsih Retnaningsih, Zenitalia Pasongka, Flora Eka Sari, Basuki Rachmad

**Affiliations:** 1 Stem Cell and Cancer Research (SCCR), Faculty of Medicine, Universitas Islam Sultan Agung (Unissula), Semarang, Central Java, Indonesia; 2 Department of Postgraduate Biomedical Science, Faculty of Medicine, Universitas Islam Sultan Agung (Unissula), Semarang, Central Java, Indonesia; 3 Department of Pathological Anatomy, Faculty of Medicine,, Universitas Islam Sultan Agung (Unissula), Semarang, Central Java, Indonesia; 4 Department of Internal Medicine, Faculty of Medicine, Universitas Muhammadiyah Yogyakarta, Yogyakarta, Yogyakarta, Indonesia; 5 Department of Molecular Biology, Faculty of Medicine, Universitas Katholik (Unika) Soegijapranata, Semarang, Central Java, Indonesia; 6 Department of Internal Medicine, Bhayangkara Hospital, Makassar, South Sulawesi, Indonesia; 7 Department of Internal Medicine, Dr. Esnawan Antariksa Air Force Hospital, Jakarta, Jakarta, Indonesia; 8 Department of Neurology and Intensive Care Unit, Kariadi Hospital, Universitas Diponegoro, Semarang, Central Java, Indonesia; 9 Faculty of Medicine, Universitas Udayana, Denpasar, Bali, Indonesia; 10 Department of Pulmonary Medicine, Dr. Esnawan Antariksa Air Force Hospital, Jakarta, Jakarta, Indonesia; 11 Department of Intensive Care Unit, Gatot Soebroto Army Hospital, Jakarta, Jakarta, Indonesia

**Keywords:** COVID-19, secretome, mesenchymal stem cells

## Abstract

Severe acute respiratory syndrome coronavirus 2 (SARS-CoV-2) is responsible for the outbreak of coronavirus disease 2019 (COVID-19), which has been rapidly spreading. Several guideline therapies have been proposed as a possible treatment for SARS-CoV-2, however, these therapies are not sufficient to treat a severe condition of SARS-CoV-2 infection characterised by the increase of D-dimer and C-reactive protein (CRP) levels, and patchy ground-glass opacities (GGOs). Secretome-mesenchymal stem cells (S-MSCs) produced by MSCs under hypoxia could excessively release several anti-inflammatory cytokines and growth factors to control the COVID-19 cytokine storm and accelerate lung injury improvement. This is the first study investigating the clinical outcomes of three severe COVID-19 patients admitted to the intensive care unit of three different hospitals in Indonesia treated with S-MSCs. The decrease of D-dimer and CRP level was reported for all patients treated with S-MSCs. This was in line with improvement of pulmonary radiology, blood gas level, and hematologic assessment. In conclusion, these cases suggest that S-MSCs could effectively control D-dimer, CRP level and GGOs of severe COVID-19 patients associated with recovered pulmonary function.

## Introduction

Since December 2019, severe acute respiratory syndrome corona virus 2 (SARS-CoV-2), responsible for the outbreak of coronavirus disease 2019 (COVID-19), has been rapidly spreading worldwide ^1^. The number of infected persons has exceeded 87 million with 2 million deaths globally ^2^. Several guideline therapies such as remdesivir and convalescent plasma have been proposed as possible treatment for SARS-CoV-2, however these treatments remain controversial. Moreover, these therapies were not effective to treat severe infection of SARS-CoV-2 due to these treatments potentially inducing the robust cytokine storm ^3, 4^. A previous study demonstrated that there is a correlation between disease severity and the release of proinflammatory cytokines, such as tumor necrosis factor-α (TNF-α), IL-6, IL-1B, IL-4, IFN-γ, IFN-γ-induced protein 10 (IP10), monocyte chemoattractant protein-1 (MCP-1), macrophage inflammatory protein-1a (MIP-1a), and granulocyte-colony stimulating factor (G-CSF) ^5^. This finding was confirmed by the high plasma cytokines found in the most severe COVID-19 patients associated with extensive lung damage ^6, 7^. Therefore, finding an effective therapeutic option to hamper the devastating cytokine storm of COVID-19 and regenerate the damaged lung is crucial. Previous studies recently reported several benefits of mesenchymal stem cells (MSCs) under hypoxia condition to inhibit robust proinflammatory cytokines and repair extensive tissue damage by releasing several anti-inflammatory cytokines and growth factors ^8^.

The use of hypoxia-MSCs (H-MSCs) could become an alternative solution to treat the severe cytokine storm of COVID-19. A previous study reported that hypoxia precondition treated on MSCs (H-MSCs) could enhance their survival to reach the damaged area ^9^. However, blood clots appearing during the severe phase of COVID-19 could block the H-MSCs trajectory into the damaged area ^10^. Other studies reported that H-MSCs could enhance the release of their active soluble molecules, known as secretome-MSCs (S-MSCs) such as IL-10, TGF beta, VEGF and PDGF, which are useful in hampering inflammation and improving tissue healing ^11^. Therefore, isolating and concentrating the exact active soluble molecule of S-MSCs is a possible strategy to control the cytokine storm of COVID-19, and, in addition, to accelerate the damaged lung improvement.

In a recent study, we successfully isolate S-MSCs from their culture medium using tangential flow filtration (TFF) strategy with several molecular weight cut-off category ^12^. In this Clinical Practice article, we report on three severe COVID-19 patients with several comorbidities who were treated with S-MSCs in three different hospitals in Indonesia. This is the first report to describe the complete monitoring of these three patients.

## Ethical considerations

Ethical clearance for the use of S-MSCs in COVID-19 cases and the protocol for administration was obtained from the Health Research Ethics Committee of Bethesda Hospital, Yogyakarta, Indonesia (approval number, No.91/KEPK-RSB/VI/20). Written informed consent for treatment with S-MSCs was obtained from each patient prior to treatment. All patients were treated with standard treatments for severe condition of COVID-19 infection, in addition to novel S-MSCs therapy regarding. Each patient was treated with three, four and six doses of 1 mL S-MSCs every 12 h (with molecular weight cut-off combination of 10–50 kDa 50%, 50–100 kDa 25%, and 100–300 kDa 25%) via deltoid intramuscular injection, respectively. A different S-MSCs dose was utilized in the three patients due to the preliminary nature of this treatment. 

## Case reports

### Case no. 1

**Figure 1.**
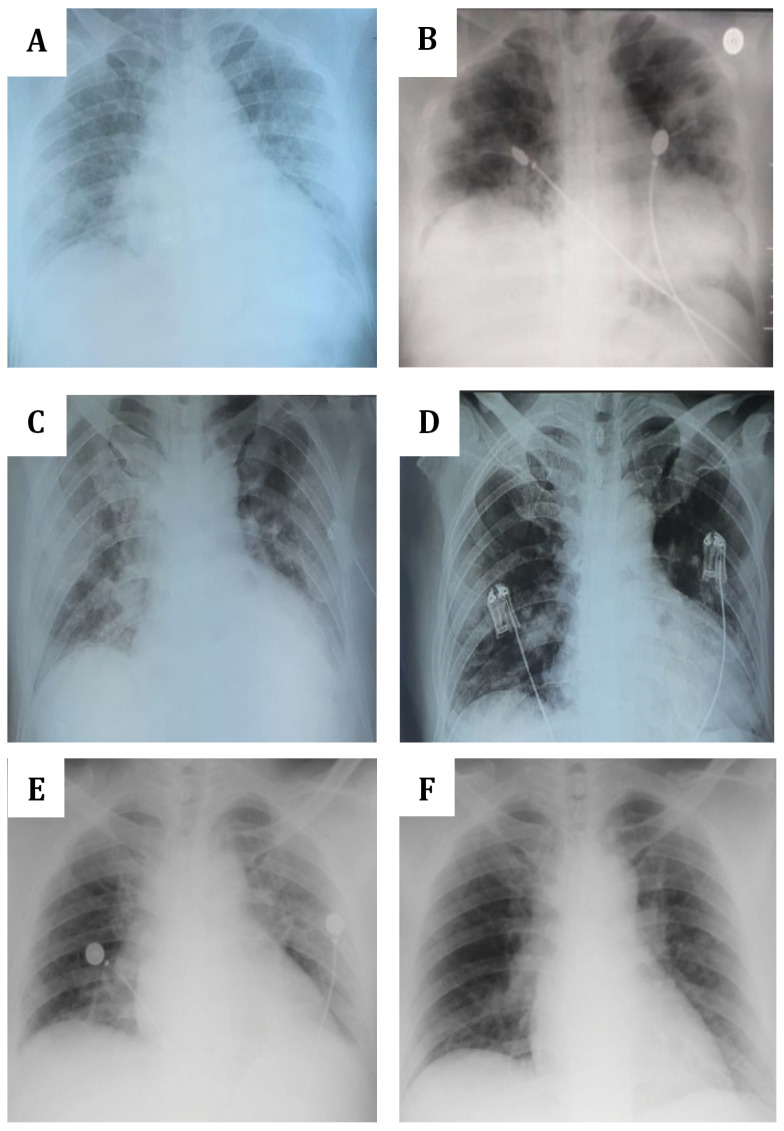
Chest X-ray of the three patients. ( **A** and **B**) *Patient 1.* ( **A**) August 29 (a day post onset of illness (dpoi), showing bronchopneumonia with bilateral ground glass opacities (GGOs) and cardiomegaly; ( **B**) September 10 (11 dpoi), showing the absorption of bilateral GGOs with no bronchopneumonia and cardiomegaly. ( **C** and **D**) *Patient 2*. ( **C**) November 19 (3 dpoi), showing cardiomegaly with lung edema, bilateral GGOs aorta elongation and aorta atherosclerosis; ( **D**) November 22 (6 dpoi), showing improvement with minimum infiltrate on pulmonalis dextra and sinistra. ( **E** and **D**) *Patient 3*. ( **C**) December 23 (7 dpoi), showing worsened bilateral GGOs with cardiomegaly and aortic atherosclerosis; ( **F**) December 28 (12 dpoi), showing decreased bilateral GGOs, cardiomegaly and aortic elongation.

A 54-year-old Indonesian male with severe hypertension was diagnosed with COVID-19 on August 28, 2020 and admitted to Dr. Esnawan Antariksa Air Force Hospital, Jakarta, Indonesia intensive care unit (ICU) with cough and dyspnea ( Table 1) and was treated with standard treatment ( Table 2). The examination showed a temperature of 36°C, a 102/min pulse, a respiratory rate of 32/min, and a blood pressure of 200/100. Blood gas analysis showed decreased oxyhemoglobin saturation (SO2, 80.6%; normal reference: 95–100%), CO _2 _partial pressure (PCO _2_, 22.9 mmHg; normal reference: 38–42 mmHg), oxygen partial pressure (PO _2_, 37.6 mmHg; normal reference: 70–99 mmHg) and HCO _3_^-^ (18.9 mmol/L; normal reference: 22–29 mmol/L). Laboratory studies showed increased white blood cells (WBC) count (17.2 × 10 ^9^ /L; normal reference: 4–10 × 10 ^9^ /L), monocyte count (9%; normal reference: 2–8%) and decreased lymphocyte count (15%; normal reference: 20–40%). On August 29, D-dimer was increased (1540 ng/mL; normal reference: 0–231 ng/mL) in line with the elevation of C-reactive protein (CRP, 61.7 mg/dL; normal reference: 0–8.1 mg/L) ( Table 3). Chest X-ray showed bronchopneumonia with bilateral ground glass opacities (GGOs) and cardiomegaly condition ( Figure 1).

**Table 1.** Clinical characteristics of COVID-19 patients receiving S-MSCs.

Patient no.	Sex	Age	Clinical classification	Days of S-MSCs therapy from symptom onset	Principal symptoms	Comorbidity
1	M	54	Severe	11	Cough, dyspnea	Severe hypertension
2	M	53	Severe	5	Cough, dyspnea, chest pain and fatigue	Type-2 diabetes mellitus
3	M	72	Severe	8	Abdominal pain, diarrhea, anosmia, cough and sore	Mild hypertension, liver failure, long-term sequelae of stroke, thalassemia minor

**Table 2.** Standard treatment received by the three patients receiving S-MSCs.

Patient no.	Drugs Administrated			Oxygen support	
	Antiviral treatment	Antibiotic or antifungal treatment	Corticosteroids treatment	Before S-MSCs Therapy	After S-MSCs therapy
1	Remdesivir 100 mg, q24h i.v. in 5 days	Azithromycin po. and Levofloxacin i.v.	Dexamethasone i.v.	High-flow nasal oxygen	nasal cannula
2	Oseltamivir 75 mg, q12h po. in 5 days	Azithromycin po. Levofloxacin i.v.	None	High-flow nasal oxygen	nasal cannula
3	Favipiravir 600mg q12h po. In 5 days	Azithromycin po., Tazobactam Sodium and Levofloxacin i.v.	Dexamethasone i.v.	High-flow nasal oxygen	nasal cannula

po., per os; i.v., i.v. injection; q12h, every 12 h; q24h, every 24 h.

**Table 3.** Comparison of laboratory parameters before and after S-MSCs treatment.

Clinical Factors	Before S-MSCs treatment			After S-MSCs treatment		
	Patient 1	Patient 2	Patient 3	Patient 1	Patient 2	Patient 3
CRP (mg/L, normal reference: 0–8.1 mg/L)	61.7	160	118	0.37	5.12	8.5
D-dimer (ng/mL, normal reference: 0–231 ng/mL)	1540	880	235	384	660	87
Lymphocyte (%, normal reference: 20–40%)	15	10	11.7	20	27	25.8
SO _2_ (%, normal reference: 95–100%)	80.6	90.6	85	99.6	95.9	98

The patient was treated with 1 mL S-MSCs three times every 12 h via deltoid intramuscular on August 30 and 31.

On September 4, SO _2_ was increased (99.6%) with increased PCO _2_ (36.2 mmHg), PO _2_ (198.7 mmHg), and HCO _3_^-^ (24.7 mmol/L). Laboratory studies showed normal WBC count (7.4 × 10 ^9^/L), monocyte count (5%) and lymphocyte count (20%). D-dimer and CRP level were decreased (1297 ng/mL and 2.33 mg/dL, respectively) ( Table 3). The chest X-ray still showed a bronchopneumonia with cardiomegaly condition ( Figure 1). On September 10, the patient was reported negative from COVID-19 infection. The chest X-ray showed improvement with no both bronchopneumonia and cardiomegaly observed. D-dimer was decreased (384 ng/mL), and CRP was normal (0.31 mg/dL). On 20 September, the patient has no cough and dyspnea. The examination showed that SO _2_ was 98.7%. He was discharged from ICU and mobilized into rehabilitation room for standard recovery of physical activity.

### Case no. 2

**Figure 2.**
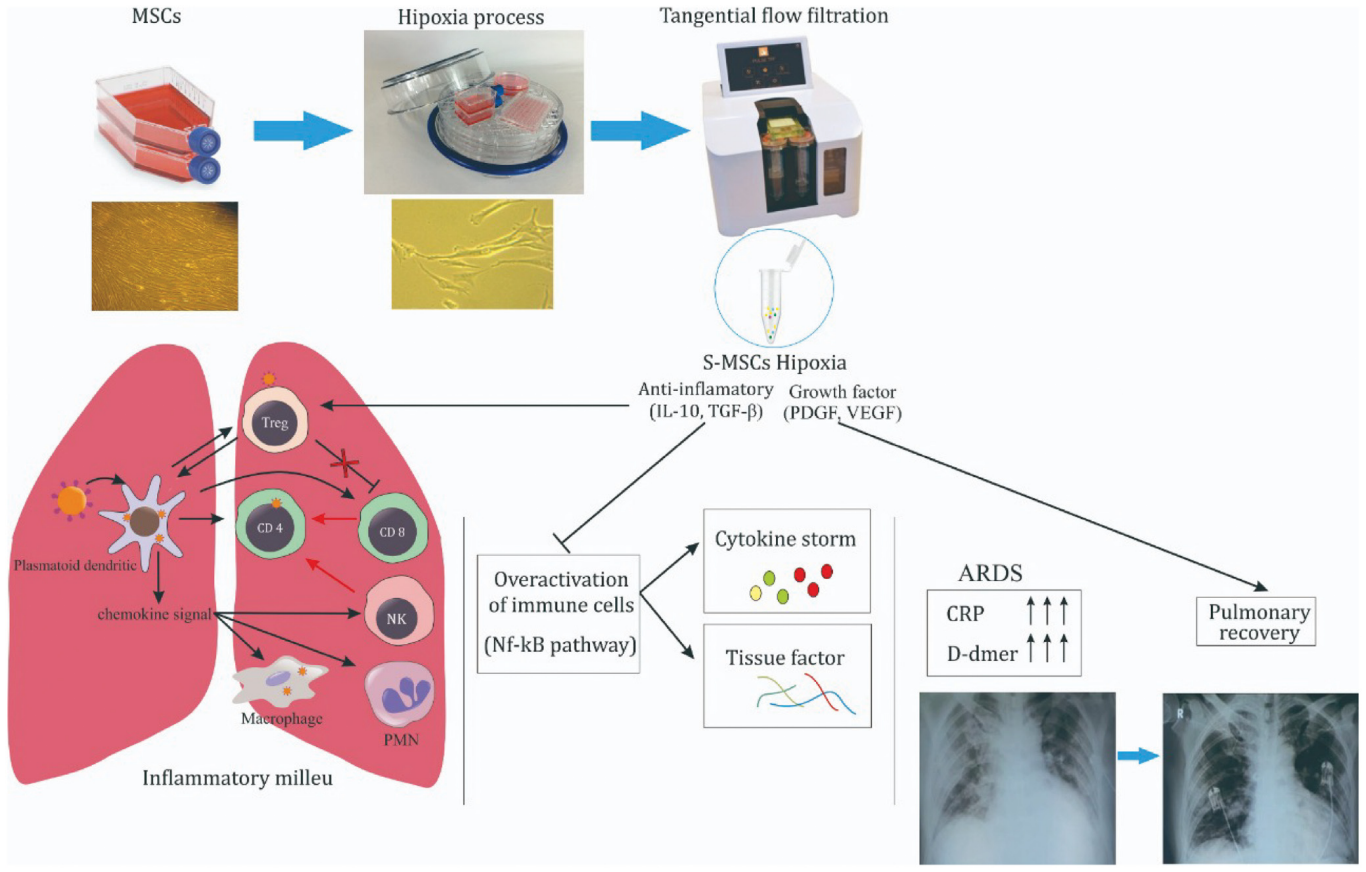
Schematic of cytokine storm on severe COVID-19 treated by S-MSCs. MSCs were incubated in 5% O _2_ hypoxia condition and S-MSCs was isolated from the culture medium using TFF technique ^12^. S-MSCs contain soluble molecules, including IL-10, TGF-β, PDGF and VEGF. IL-10 and TGF-β inhibit NF-kB pathways activation of overactivated immune cells leading to cytokine storm inhibition, characterized by the decreased level of CRP and D-dimer. Under controlled inflammatory milieu, VEGF and PDGF promote the improvement of bilateral GGOs marked by pulmonary recovery.

A 53-year-old Indonesian male with type 2 diabetes mellitus was diagnosed with COVID-19 on November 16, 2020. He was admitted to Gatot Soebroto Army Hospital, Jakarta, Indonesia on November 17 with cough, dyspnea, chest pain and fatigue ( Table 1). On November 19, he was admitted to the ICU due to worsened dyspnea and treated with standard treatment ( Table 2). Chest X-ray revealed cardiomegaly with lung edema, bilateral GGOs, aorta elongation and aorta atherosclerosis ( Figure 2). Blood gas analysis showed PCO _2_ (29.9 mmHg), PO _2 _(177.1 mmHg) and HCO _3_^-^ (20.8 mmol/L), however SO _2_ was still normal (98%). On November 21, the SO _2_ was 90.6% (abnormal) with reduced PO _2 _(57.5 mmHg). Laboratory studies showed normal WBC count (6.02 × 10 ^9^/L) with increased neutrophil count (81%, normal reference: 50–70%), monocyte count (9%) and decreased lymphocyte count (10%). D-dimer was abnormal (880 ng/mL). Blood chemistries revealed elevations in CRP (160 mg/L) and fasting plasma glucose (FPG, 398 mg/dL; normal reference: 70–140 mg/dL ( Table 3).

Due to the patient’s worsening condition, he was injected with 1 mL S-MSCs four times every 12 h via deltoid intramuscular injection on November 21 and 22. On the same day, 6 hours after injection, SO _2_ increased (98.7%) with increased PO _2_ (138.5 mmHg), pH (7.509) and HCO _3_^-^ (29.7 mmol/L). On November 22, chest X-ray showed improvement with minimum infiltrate on pulmonalis dextra and sinistra ( Figure 1). On November 23, oxygen saturation was normal (95.9%), with normal PCO _2_ (36.1 mmHg) and PO _2 _(71.5 mmHg). Laboratory studies showed increased neutrophil count (85%) with normal monocyte count (6%) and decreased lymphocyte count (9%). D-dimer was decreased (660 ng/mL). Blood chemistries revealed decreased fasting plasma glucose (277 mg/dL) and CRP (5.12 mg/dL ( Table 3). On December 5, the patient has no cough, dyspnea, chest pain and fatigue. Examination showed that SO _2_ was 99.2%. He was discharged from ICU and mobilized into rehabilitation room for physical activity recovery. On December 28, laboratory studies showed normal neutrophil (57%) and lymphocyte count (27%) with increased monocyte count (11%). Chest X-ray showed normal cardiac physiology and no infiltrate or nodule in both pulmonalis.

### Case no. 3

A 72-year-old Indonesian male with mild hypertension, liver failure, long-term sequelae of stroke and thalassemia minor was diagnosed with COVID-19 on December 16, 2020 and admitted to Bhayangkara Hospital, Makassar, Indonesia with abdominal pain, diarrhea, anosmia, cough and sore throat in the last three days ( Table 1). The examination showed a temperature of 37.3°C, pulse of 82/min, respiratory rate of 24/min, blood pressure of 140/90 mmHg, and SO _2 _of 97%. Laboratory studies showed a decreased WBC count (3.1 × 10 ^9^ /L), with normal neutrophil count (53.4%), lymphocyte count (26.8%) and increased monocyte count (18.8%). Chest X-ray showed bilateral GGOs. On December 23, the patient’s dyspnea worsened and he was admitted to the ICU and treated with standard treatment ( Table 2). Blood gas analysis revealed that oxygen saturation was decreased (85%) with decreased PCO _2_ (30 mmHg), PO _2 _(49 mmHg) and HCO _3_^-^ (19 mmol/L). Laboratory tests showed an increased neutrophil count (72.9%), monocyte count (14.9%) and decreased lymphocyte count (11.7%). D-dimer was increased (235 ng/mL) and blood chemistries revealed elevations in CRP (118 mg/L ( Table 3). Chest X-ray showed worsened bilateral GGOs with cardiomegaly and aortic atherosclerosis ( Figure 1).

On December 24–26, the patient was treated with 1 mL S-MSCs six times every 12 h via deltoid intramuscular injection in addition to other standard treatment ( Table 2). The day after the first S-MSC injection, oxygen saturation increased (98%). The examination showed a temperature of 37°C, pulse of 90/min, respiratory rate of 28/min and blood pressure of 120/80 mmHg. On December 28, examination showed a temperature of 37°C, pulse of 80/min, respiratory rate of 24/min and blood pressure of 120/80 mmHg. Oxygen saturation was normal (98%). Laboratory studies showed normal neutrophil count (78.3%) monocyte count (7.2%) and lymphocyte count (25.8%). D-dimer was decreased (86.9 ng/mL). Blood chemistries revealed decreased CRP (8.5 mg/dL ( Table 2). Chest X-ray showed decreased bilateral GGOs, cardiomegaly and aortic elongation ( Figure 1). On December 30, the patient has no abdominal pain, diarrhea, anosmia, cough and sore throat. The examination showed that SO2 was 99%. He was discharged from ICU and mobilized into rehabilitation room for standard physical activity recovery. On January 6, the patient was negative for COVID-19.

## Discussion

Severe pneumonia COVID-19 is characterized by rapid viral infection, excessive inflammatory cell infiltration and robust cytokine storm associated with an increase of D-dimer and CRP levels, resulting in acute respiratory distress syndrome (ARDS) ^13^. A previous study reported that the cytokine storm of COVID-19 was associated with an increase of several proinflammatory cytokines, including TNF-α, IL-1, IL-6, IL-17A and granulocyte macrophage colony-stimulating factor (GM-CSF) in the plasma. In line with this phenomenon, lymphocyte count in severe COVID-19 patients' peripheral blood was decreased ^14, 15^. Another study also revealed that lymphocyte count in severe COVID-19 patients was reduced due to overactivated immune cells, known as macrophage activated syndrome (MAS). The MAS potentially promotes the excessive cytokines storm characterized by the increase of CRP and D-dimer level leading to bilateral GGOs ^16^. S-MSCs contain several anti-inflammatory cytokines, including IL-10 and TGF-β, to control the overactivated immunity and hamper the storm's excessive cytokines. In addition, S-MSCs also have several growth factors, such as VEGF and PDGF, that could accelerate lung injury improvement in COVID-19 patient ^8, 11^. 

MSCs were isolated from umbilical cord and exhibited typical monolayers of spindle-shaped fibroblast-like cells with plastic adherent capability under standard culture condition. The cultured MSCs have a high level of surface antigens, including CD90, CD73 and CD105, and lacked the expression CD34, CD45, CD11b, CD19, and HLA-DR, represented as Lin. On the other hand, they also showed the capability to differentiate into osteocyte and adipocyte (see extended data, figure S1 for more details). To induce hypoxia condition, the MSCs were treated under 5% O _2_ condition in a hypoxia incubation chamber for 24 h. After incubation, the culture medium of H-MSCs was collected and filtered using TFF strategy with molecular weight cut-off of 10–50 kDa, 50–100 kDa, and 100–300 kDa to produce the S-MSCs ( Figure 2). This strategy was employed to obtain the desired cytokines and growth factors in that range, such as IL-10, TGF-β, VEGF and PDGF. In this study, we used S-MSCs with the combination of molecular weight cut-off of 10–50 kDa (50%), 50–100 kDa (25%), and 100–300 kDa (25%). The analysis of cytokines and growth factors level on this S-MSCs combination has been employed using flow cytometry CBA detection and showed a feasible result (see extended data, table S1 for more details).

This is the first report that suggests the feasibility of S-MSC therapy of three severe COVID-19 patients in Indonesia. All patients showed bilateral GGOs on chest X-ray before treatment. The hematologic findings showed elevated D-dimer and CRP level in addition to lymphocytopenia and decrease of SO _2_ and PO _2_. These patients received several doses of S-MSCs (1 mL/dose) / 12 h which results in favorable outcomes. This study showed that between three and six doses of S-MSCs were well tolerated by the patients. The clinical symptoms were significantly improved with patchy GGO improvement, associated with the decrease of D-dimer and CRP level and increase of SO _2_ and PO _2_. From our observation, we would suggest that six doses of S-MSCs performed the most optimal treatment in the COVID-19 patients. These results suggest that the immune system's excessive inflammation and overactivation were alleviated by anti-inflammatory cytokines contained in S-MSCs, while the high level of growth factors in S-MSCs could also accelerate the improvement of GGOs. Based on our preliminary results, S-MSC therapy could be a promising and safe rescue option to treat severe COVID-19 patients.

The first key factor associated with effective S-MSC therapy is the controlled immune system overactivation resulting in the alleviated cytokines storm characterized by the decrease in CRP and D-dimer levels. In our observations, the level of CRP and D-dimer was decreased in all patients treated with S-MSCs. This data suggest that S-MSCs could effectively control the overactivated immune cells. A previous study reported that S-MSCs could control proinflammatory immune cells due to their anti-inflammatory cytokines, such as IL-10 and TGF-β ^11, 17^. IL-10 could hamper the inflammatory cells' activity by activating tyrosine kinase-2 and Janus tyrosine kinase 1 (JAK1) and inhibiting NF-kB pathways leading to the decreased expression of proinflammatory cytokines such as TNF-α, IL-1, IL-6 and IL-17A ^9, 18^. On the other hand, TGF-β could activate the regulatory subset of T lymphocyte (Treg) by initiating the FoxP3 expression, resulting in suppressing overactivated immune cells ^19^ ( Figure 2).

Another key factor associated with the efficacy of S-MSCs treatment is the acceleration of lung injury improvement characterized by the improvement pulmonary radiological results, decrease of CRP and D-dimer level resulting in the controlled inflammation leading to normal pulmonary function marked by normal SO _2_. In our patients, decreased CRP and D-dimer was associated with the improvement of patchy GGOs and normal SO _2_ in all patients treated with S-MSCs. These data suggest that along with the controlled immune overactivation, the growth factors contained in S-MSCs could rapidly improve lung regeneration post excessive cytokine storm exposure. A previous study revealed that S-MSCs could accelerate wound healing due to their growth factors, particularly VEGF and PDGF ^20^. VEGF and PDGF could accelerate the repair of leaky pulmonary blood vessels and accelerate lung injury improvement through the MEK and Akt pathway resulting in the acceleration of angiogenesis and reduced pulmonary infiltrate characterized by decreased bilateral GGOs ^21, 22 ^( Figure 2).

In our patients, no severe adverse effects were observed. However, one limitation of our report is that the dynamic changes of cytokine and growth factors during treatment were not investigated. Nevertheless, the preliminary result seem promising.

In conclusion, we report that S-MSC therapy shows a potential therapeutic effect and low risk in severe COVID-19 patient treatment. We observed that between three and six doses of S-MSCs, with weight cut-off combination of 10–50 kDa (50%), 50–100 kDa (25%) and 100–300 kDa (25%), rapidly controlled the excessive cytokine storm in our patients and improved lung injury. Six doses showed optimum outcome. The treatment time point and the clear clinical advantages of S-MSCs therapy need to be further investigated in randomized clinical studies.

## Consent

Written informed consent for publication of this case report, along with any associated images, was obtained from all three patients.

Written informed consent was also obtained from the patients to undergo treatment using the novel treatment.

## Data availability

### Extended data

Open Science Framework: Secretome H-MSCs for COVID-19 extended data. https://doi.org/10.17605/OSF.IO/RJ8PC

Data are available under the terms of the Creative Commons Zero "No rights reserved" data waiver (CC0 1.0 Public domain dedication).
