# Supplementary material for: Case series of the first three severe COVID-19 patients treated with the secretome of hypoxia-mesenchymal stem cells in Indonesia
Source: F1000Res. 2021 Oct 22;10:228. Originally published 2021 Mar 22. [Version 3] doi: 10.12688/f1000research.51191.3 (PMC8927740; doi:10.12688/f1000research.51191.3)
Supplement: Peer review history

# Peer review: Reviewer response for version 3

**DOI:** 10.5256/f1000research.78517.r97656

**Published:** 2022-03-16

**Authors:** Rajesh Ramasamy

**Affiliations:** 1 Department of Pathology, Universiti Putra Malaysia, Selangor, Malaysia

The authors have provided clarifications regarding the requested technical details. Although the reported clinical research is not a comprehensive clinical trial, the use of MSCs in 3 clinical cases brought insight to further exploring the mesenchymal stem cell therapy for severe lung-injured COVID-19 patients.

Are enough details provided of any physical examination and diagnostic tests, treatment given and outcomes?

Partly

Is the background of the cases’ history and progression described in sufficient detail?

Yes

Is sufficient discussion included of the importance of the findings and their relevance to future understanding of disease processes, diagnosis or treatment?

Yes

Is the conclusion balanced and justified on the basis of the findings?

Partly

Reviewer Expertise:

Mesenchymal Stem Cell and Immunomodulation

I confirm that I have read this submission and believe that I have an appropriate level of expertise to confirm that it is of an acceptable scientific standard.

# Peer review: Reviewer response for version 2

**DOI:** 10.5256/f1000research.58709.r90165

**Published:** 2021-09-10

**Authors:** Rajesh Ramasamy

**Affiliations:** 1 Department of Pathology, Universiti Putra Malaysia, Selangor, Malaysia

The authors have submitted an acceptable characterisation of MSCs; however, the characterisation data that include immunophenotyping and mesodermal differentiation would be complete when:

1. chondrogenic differentiation data is included. A complete mesodermal differentiation based on acceptable standards comprises osteo, adipo and chondro differentiations;  
2. it has provided isotype or unstained control for lineage negative staining. As a practice, the negative population is usually located within the first quarter or first half of the antibody axis. In the current supplementary data, the negative population has been depicted in the centre of the antibody axis.

Are enough details provided of any physical examination and diagnostic tests, treatment given and outcomes?

Partly

Is the background of the cases’ history and progression described in sufficient detail?

Yes

Is sufficient discussion included of the importance of the findings and their relevance to future understanding of disease processes, diagnosis or treatment?

Yes

Is the conclusion balanced and justified on the basis of the findings?

Partly

Reviewer Expertise:

Mesenchymal Stem Cell and Immunomodulation

I confirm that I have read this submission and believe that I have an appropriate level of expertise to confirm that it is of an acceptable scientific standard, however I have significant reservations, as outlined above.

# Response

**Published:** 2021-10-02

**Authors:** Agung Putra

**Affiliations:** Stem Cell and Cancer Research Indonesia, Indonesia

Dear Reviewer:

 Thank you for reviewing our manuscripts, entitled “Case series of the first three severe COVID-19 patients treated with the secretome of hypoxia-mesenchymal stem cells in Indonesia”. We appreciate the time and effort that reviewers dedicated to providing some feedback on our manuscript. We have incorporated most of the suggestions made by the reviewers. 

 Reviewer Comments:

 "The authors have submitted an acceptable characterization of MSCs; however, the characterization data that include immunophenotyping and mesodermal differentiation would be complete when:

- chondrogenic differentiation data is included. A complete mesodermal differentiation based on acceptable standards comprises osteo, adipo and chondro differentiations; "

**Author response:**

 Analysis of the differentiation capability of MSCs into 3 mesodermal tissues according to the guidelines is actually needed. However, the completeness of the differentiation analysis is not mandatory. We refer to several articles related to the study of MSCs regarding the incompleteness of the differentiation analysis data:

 1. Lotfinia M, Lak S, Mohammadi Ghahhari N, et al. Hypoxia Pre-Conditioned Embryonic Mesenchymal Stem Cell Secretome Reduces IL-10 Production by Peripheral Blood Mononuclear Cells. Iran Biomed J. 2017;21(1):24-31. doi:10.6091/.21.1.24

 2. Jeske R, Yuan X, Fu Q, Bunnell BA, Logan TM, Li Y. In Vitro Culture Expansion Shifts the Immune Phenotype of Human Adipose-Derived Mesenchymal Stem Cells. Front Immunol. 2021;12:621744. Published 2021 Mar 10. doi:10.3389/fimmu.2021.621744

 3. Qu X, Liu X, Cheng K, Yang R, Zhao RC. Mesenchymal stem cells inhibit Th17 cell differentiation by IL-10 secretion. Exp Hematol. 2012;40(9):761-770. doi:10.1016/j.exphem.2012.05.006

 However, the lack of MSCs differentiation data analysis remains a limitation of our study in which we will improve in further studies.

- "It has provided isotype or unstained control for lineage negative staining. As a practice, the negative population is usually located within the first quarter or first half of the antibody axis. In the current supplementary data, the negative population has been depicted in the center of the antibody axis."

**Author response:**

 The isotype for lineage negative staining has been revised and included in the new extended data files. Reviewers could check our revised extended data on https://doi.org/10.17605/OSF.IO/RJ8PC

# Peer review: Reviewer response for version 1

**DOI:** 10.5256/f1000research.54326.r87539

**Published:** 2021-07-05

**Authors:** Guido Moll

**Affiliations:** 1 BIH Center for Regenerative Therapies (BCRT) and Berlin-Brandenburg School of Regenerative Therapies (BSRT), Berlin Institute of Health, Charité - University Medicine Berlin, Berlin, Germany

In this Case Study, Putra *et al.* report the experimental treatment of three patients suffering from severe COVID-19 with hypoxia-conditioned mesenchymal stromal cell (MSC)-derived secretome (S-MSC) prepared by tangential flow filtration (TFF), to prepare the highly concentrated/enriched MSC secretome for clinical application. In their introduction and discussion, the authors identify among others the COVID-19 cytokine storm and concomitant severe lung and tissue damage, as targets of MSC therapy, anticipating that MSC therapy may exert pleiotropic beneficial effects.

 The Case Study was conducted at Bethesda Hospital in Yogyakarta, Indonesia (With Local Health Research Committee approval number No.91/KEPK-RSB/V1/20), with written informed consent for the treatment with S-MSCs obtained from each patient prior to treatment. All patients received standard treatment for severe COVID-19, with on-top adjunct treatment with S-MSCs. This is a Case Report of three patients, who were all treated in exploratory fashion with different doses of S-MSCs, and there is no control group or control patients reported in the study.

 The S-MSC was prepared by TFF to enrich bioactive factors secreted by the MSCs into 1ml therapeutic doses. The S-MSCs were applied in each of the three patients six times every 12 hours via deltoid intramuscular injection. Molecular weight cut-off combination of 10-50 kDa 50%, 50-100 kDa 25%, and 100-300 kDa 25% were used.  The production method of S-MSC is outlined in reference number 12: Haraszti *et al.* ^1^ Exosomes Produced from 3D-cultures of MSCs by Tangential Flow Filtration Show higher Yield and Improved Activity published in Molecular Therapy in 2018.

 The authors report:

- In Table 1) Clinical Characteristics of Patients Receiving S-MSC: Patient Sex, Age, Clinical Classification (Severe COVID-19), Principal Symptoms, Respective Comorbidities (e.g. Hypertension, T2D, Liver Failure, Stroke, Thalassemia);
- In Table 2) Standard Treatment Received by the Three Patients: Drugs Administered (Antiviral, Antibiotic, Antifungal, and Corticosteroid Treatment) and Oxygen Support (Before / After S-MSC Therapy);
- In Table 3) Comparison of Laboratory Parameters Before and After S-MSC Treatment (CRP, D-Dimer, Lymphocytes, SO2); and in Figure 1 and 2) Chest X-ray’s of the Three Patients and a Schematic Study Overview, respectively.

Given the standard of care treatment, that there was no control group, and that this was an open label Case Study, the key findings of this report are as follows (although any of these findings could also be a result of the standard treatment):

1. CRP was strongly log-scale reduced after S-MSC treatment (from 62, 160, 118 to 0.4, 5.1, and 8.5),
2.  D-dimer was reduced around 2-fold on average (1540, 880, 235 to 384, 660, 87),
3.  Lymphocyte counts normalized/2-fold increased on average (15, 10, 12 to 20, 27, 26),
4.  SO2 increased/improved (89.6, 90.6, 85 to 99.6, 95.9, 98) together with PO2, and 5) Chest x-rays mainly indicated that lung pathology and cardiomegaly improved (reduction of bilateral ground glass opacities and cardiomegaly).

In their introduction and discussion, the authors briefly outline the study hypothesis and why MSC / S-MSC treatment may be beneficial and relate this to the improved functional parameters in their Case Study, and these findings are in line with other clinical reports on MSC treatment of COVID-19. So it stands to reason that hypoxia conditioned S-MSC (concentrated soluble fraction of MSC media) is first of all safe, and second may be just as efficient as cellular MSCs in treating COVID-19. However, given the lack of a control group, and open-label treatment of these three patients the findings should be interpreted with caution. Interestingly, not only inflammation and lung parameters improved, but also D-dimer levels, as typically observed in the follow-up of successfully treated patients, see “Viscoelastic Testing Reveals Normalization of the Coagulation Profile 12 Weeks After Severe COVID-19” ^2^ published in Scientific Reports in 2021.

 The authors recognized COVID-19 coagulopathy as a challenge for MSC therapy, as reported earlier in “MSC Therapies for COVID19: Importance of Patient Coagulopathy, Thromboprophylaxis, Cell Product Quality and Mode of Delivery for Treatment Safety and Efficacy” ^3^ published in Frontiers in Immunology 2020 and “Intravascular MSC Therapy Product Diversification: Time for New Clinical Guidelines” ^4^ published in Trends in Molecular Medicine 2019). Another crucial point is the appropriate timing and dosing and mode of application of MSC therapy in COVID-19, "Mesenchymal Stromal Cell Therapy for Coronavirus Disease 2019: Which? When? And How Much?" ^5^ published in Cytotherapy 2021.

 The authors indicate their approach as a rescue treatment for patients progressed to severe COVID-19 stage (often presenting with pronounced coagulopathy). Here, the application of cellular secretome instead of the actual cellular MSC products may be somewhat superior/safer given the expression of varying levels of highly procoagulant Tissue Factor (TF/CD142) on MSCs (See PMID’s 32574263 ^3^ and 30711482 ^4^. In this context, another key advantage of this study may be that the S-MSC was applied via deltoid intramuscular injection.

 Future studies should also put more emphasis on well-controlled dose escalation, pharmacokinetics and pharmacodynamics, as well as biodistribution of the product with tight monitoring of coagulation/complement markers and other safety parameters, adjunct with deeper monitoring of a broader panel of biomarkers of response (e.g. systemic and potentially tissue cytokine profiling in blood and BAL as well as longitudinal monitoring of immune cell subsets in the periphery and local tissue BAL). Another important point is the timing of the treatment relative to the diseases stage (e.g. newly diagnosed vs. progressed patients, identify optimal treatment window). Presumably, S-MSC is an allogeneic off-the-shelf product, thus aspects of alloimunization should be studied in more detailed (humoral and cellular alloimunization). Another important issue is the GMP-compliant production of S-MSC for use in human patients.

Are enough details provided of any physical examination and diagnostic tests, treatment given and outcomes?

Yes

Is the background of the cases’ history and progression described in sufficient detail?

Yes

Is sufficient discussion included of the importance of the findings and their relevance to future understanding of disease processes, diagnosis or treatment?

Yes

Is the conclusion balanced and justified on the basis of the findings?

Yes

Reviewer Expertise:

Immunology; Cardiovascular System; COVID-19; Stem Cell Therapy; Mesenchymal Stromal Cells (MSCs)

I confirm that I have read this submission and believe that I have an appropriate level of expertise to confirm that it is of an acceptable scientific standard.

## References

[1] : Exosomes Produced from 3D Cultures of MSCs by Tangential Flow Filtration Show Higher Yield and Improved Activity. *Molecular Therapy* .2018;26(12) : 10.1016/j.ymthe.2018.09.015 2838-2847 10.1016/j.ymthe.2018.09.015 PMC627755330341012

[2] : Viscoelastic testing reveals normalization of the coagulation profile 12 weeks after severe COVID-19. *Scientific Reports* .2021;11(1) : 10.1038/s41598-021-92683-1 10.1038/s41598-021-92683-1 34172793PMC8233385

[3] : MSC Therapies for COVID-19: Importance of Patient Coagulopathy, Thromboprophylaxis, Cell Product Quality and Mode of Delivery for Treatment Safety and Efficacy. *Front Immunol* .2020;11: 10.3389/fimmu.2020.01091 1091 10.3389/fimmu.2020.01091 32574263PMC7249852

[4] : Intravascular Mesenchymal Stromal/Stem Cell Therapy Product Diversification: Time for New Clinical Guidelines. *Trends Mol Med* .25(2) : 10.1016/j.molmed.2018.12.006 149-163 10.1016/j.molmed.2018.12.006 30711482

[5] : Mesenchymal stromal cell therapy for coronavirus disease 2019: which? when? and how much?. *Cytotherapy* .2021; 10.1016/j.jcyt.2021.04.004 10.1016/j.jcyt.2021.04.004 34053857PMC8084615

# Response

**Published:** 2021-07-17

**Authors:** Agung Putra

**Affiliations:** Stem Cell and Cancer Research Indonesia, Indonesia

Dear Reviewer

 Thank you for giving us the opportunity to submit a revised of our manuscripts “Case series of the first three severe COVID-19 patients treated with the secretome of hypoxia-mesenchymal stem cells in Indonesia”. We appreciate the time and effort that reviewers dedicated to providing feedback on our manuscript and are grateful for the insightful comments on and valuable improvements to our paper. We have incorporated most of the suggestions made by the reviewers. Those changes are highlighted within the manuscript and extended data. Please see below, in bold, for a point-by-point response to the reviewers’ comments and concerns. All page numbers refer to the revised manuscript file with tracked changes (yellow highlight).

  

- This is a Case Report of three patients, who were all treated in exploratory fashion with different doses of S-MSCs, and there are no control group or control patients reported in the study.

**Author response: Thank you for pointing this out. The reviewer is correct that we are reporting the findings of a case report study, and we do not utilize a control group to compare the relevance of the results.**

  

- So, it stands to reason that hypoxia conditioned S-MSC (concentrated soluble fraction of MSC media) is first of all safe,

**Author response: We confirm the reviewer's statement if certainly what was emphasized at the outset was the safety of the use of S-MSCs. We believe that S-MSC is safe because it has undergone a sterile ultrafiltration process using a TFF strategy of up to 10 kDA in size, so contamination is not possible. Future studies of S-MSCs will lead the safety parameters.**

  

- Second maybe just as efficient as cellular MSCs in treating COVID-19.

**Author Response: Regarding the efficacy of S-MSCs to treat the severe condition of COVID-19, we suggest that the S-MSCs is more efficient than cellular MSCs. This is due to the pulmonary intravascular coagulopathy (PIC) that present in severe COVID-19 patients could blockage the direction of cellular MSCs to homing to injured pulmonary. We use S-MSCs molecules under 300 kDA which makes it possible to get past the blockages due to PIC to modulate the inflammatory condition and enhance pulmonary regeneration.**

  

- Interestingly, not only inflammation and lung parameters improved, but also D-dimer levels, as typically observed in the follow-up of successfully treated patients

**Author response: We suggest that the possibility of S-MSCs to improve D-dimer levels, besides lung parameters is the controlled immune system's excessive inflammation and overactivation and the improvement of the lung caused by anti-inflammatory cytokines and growth factors contained in S-MSCs which possible to pass the PIC blockages.**

  

- Future studies should also put more emphasis on well-controlled dose-escalation, pharmacokinetics, and pharmacodynamics, as well as biodistribution of the product with tight monitoring of coagulation/complement markers and other safety parameters, adjunct with deeper monitoring of a broader panel of biomarkers of response (e.g. systemic and potentially tissue cytokine profiling in blood and BAL as well as longitudinal monitoring of immune cell subsets in the periphery and local tissue BAL). Another important point is the timing of the treatment relative to the disease's stage (e.g. newly diagnosed vs. progressed patients, identify optimal treatment window).

**Author response: We agree that this is a potential limitation of the study. In our future study, we will analyze the pharmacokinetics, pharmacodynamics, and biodistribution of the secretome. Furthermore, in the prospective study, we will also evaluate the parameter related to cytokine profiling in blood and BAL and monitoring of immune cell subsets in the periphery and local tissue BAL. We also agree that another potential limitation is no information about the disease stage of patients receiving secretome MSCs treatment. In the future study, it is planned to make several groups of patients who describe the different disease stages to determine the optimal treatment window.**

  

- S-MSC is an allogeneic off-the-shelf product, thus aspects of alloimmunization should be studied in more detail (humoral and cellular alloimmunization). Another important issue is the GMP-compliant production of S-MSC for use in human patients

**Author response: Thank you for pointing this out. We agree that this is an important consideration, we have added the MSCs characterization in extended data, figure S1. Based on this data, the expression CD34, CD45, CD11b, CD19, and HLA-DR are represented as Lin <2%. It can be concluded that the possibility of an allergic reaction is quite limited. Although the potential of allergic reactions is limited, further studies need to be carried out.**

# Peer review: Reviewer response for version 1

**DOI:** 10.5256/f1000research.54326.r85411

**Published:** 2021-07-05

**Authors:** Rajesh Ramasamy

**Affiliations:** 1 Department of Pathology, Universiti Putra Malaysia, Selangor, Malaysia

The authors have tested the usefulness of mesenchymal stem/stromal cells-derived filtered supernatant to treat severe ARS due to COVID-19. The outcomes of the intervention were measured through the parameter of CRP, D-Dimer, and improvement of patients' clinical conditions. The tested treatment indeed showing a range of positive outcomes on the patients. The following technical queries require clarifications:

1.  Provided the supplementary laboratory data on the characterisation of MSC.
2.  Include the details of the hypoxic conditions that catered to the MSC. 
3. What would be the rationale for selecting various percentages and molecular weight for filtering the supernatant? 
4. Any profiling of cytokines or growth factors had been conducted prior to injecting the secretome into the patients?

The discussion should be made with achieved results rather than the anticipated immunosuppressive action of MSCs. There is no standardisation of MSC secretome. It could be possible that the exosome and microRNA in the secretome mediate the reparative function of MSCs through variable actions that include tissue repair.

Are enough details provided of any physical examination and diagnostic tests, treatment given and outcomes?

Partly

Is the background of the cases’ history and progression described in sufficient detail?

Yes

Is sufficient discussion included of the importance of the findings and their relevance to future understanding of disease processes, diagnosis or treatment?

Yes

Is the conclusion balanced and justified on the basis of the findings?

Partly

Reviewer Expertise:

Mesenchymal Stem Cell and Immunomodulation

I confirm that I have read this submission and believe that I have an appropriate level of expertise to confirm that it is of an acceptable scientific standard, however I have significant reservations, as outlined above.

# Response

**Published:** 2021-07-17

**Authors:** Agung Putra

**Affiliations:** Stem Cell and Cancer Research Indonesia, Indonesia

Dear Reviewer

 Thank you for giving us the opportunity to submit a revised of our manuscripts “Case series of the first three severe COVID-19 patients treated with the secretome of hypoxia-mesenchymal stem cells in Indonesia”. We appreciate the time and effort that reviewers dedicated to providing feedback on our manuscript and are grateful for the insightful comments on and valuable improvements to our paper. We have incorporated most of the suggestions made by the reviewers. Those changes are highlighted within the manuscript and extended data. Please see below, in bold, for a point-by-point response to the reviewers’ comments and concerns. All page numbers refer to the revised manuscript file with tracked changes (yellow highlight).

  

- Provided supplementary laboratory data on the characterization of MSC.

**Author response: Thank you for your suggestion. We have added the suggestion content on extended data, figure S1.**

  

- Include the details of the hypoxic conditions that catered to the MSCs.

**Author response: Thank you for this suggestion. We have added the details of hypoxic conditions on the discussion section that mark as the yellow highlight.**

  

- What would be the rationale for selecting various percentages and molecular weight for filtering the supernatant?

**Author response: We selected the 10-50 and 50-100 kDa molecular weight filtering to obtain the desired cytokines and growth factors, such as IL-10, TGF-β, VEGF, and PDGF. We also use 25% S-MSCs with 100-300 kDA categories to obtain the miRNA and exosome. However, we do not analyze the exosome and miRNA contained in S-MSCs. Further studies need to be carried out.**

  

- Any profiling of cytokines or growth factors had been conducted prior to injecting the secretome into the patients?

**Author response: We have evaluated the cytokine and growth factor profiles before injecting secretome MSCs into patients. We are confirming that the quality of the secretome MSCs administered is consistent on all three patients (See extended data, table S1 for more details).**

  

- The discussion should be made with achieved results rather than the anticipated immunosuppressive action of MSCs. There is no standardisation of MSC secretome. It could be possible that the exosome and microRNA in the secretome mediate the reparative function of MSCs through variable actions that include tissue repair.

**Author response: Thank you for your suggestion. Accordingly, throughout the manuscript, we have revised and discussed the results in the discussion section such as:**

**“The clinical symptoms were significantly improved with patchy GGO improvement, associated with the decrease of D-dimer and CRP level and increase of SO 2 and PO 2. From our observation, we would suggest that six doses of S-MSCs performed the most optimal treatment in the COVID-19 patients. These results suggest that the immune system's excessive inflammation and overactivation were alleviated by anti-inflammatory cytokines contained in S-MSCs, while the high level of growth factors in S-MSCs could also accelerate the improvement of GGOs.”**

**“In our observations, the level of CRP and D-dimer was decreased in all patients treated with S-MSCs. This data suggest that S-MSCs could effectively control the overactivated immune cells. A previous study reported that S-MSCs could control proinflammatory immune cells due to their anti-inflammatory cytokines, such as IL-10 and TGF-β ^11, 17^.”**

**We also agree with the reviewer’s assessment that there is no standardization of secretome MSCs. Secretome MSCs contain exosome and soluble molecule active. This is a potential limitation of the study because we have not standardized the secretome MSCs. Therefore, further studies of secretome MSCs standardization need to be carried out.**
